# Hydrosurgical and conventional debridement of burns: randomized clinical trial

**DOI:** 10.1093/bjs/znab470

**Published:** 2022-03-03

**Authors:** Catherine M. Legemate, Kelly A. A. Kwa, Harold Goei, Anouk Pijpe, Esther Middelkoop, Paul P. M. van Zuijlen, Gerard I. J. M. Beerthuizen, Marianne K. Nieuwenhuis, Margriet E. van Baar, Cornelis H. van der Vlies, HyCon Study Group, J. Dokter, K. L. M. Gardien, J. Hiddingh, H. W. C. Hofland, Y. Lucas, A. Meij-de Vries, T. H. J. Nijhuis, I. M. M. H. Oen, D. T. Roodbergen, S. M. H. J. Scholten-Jaegers, M. Stoop, M. Vlig, N. Trommel

**Affiliations:** 1 Burn Centre, Maasstad Hospital, 3079 DZ Rotterdam, The Netherlands; 2 Department of Plastic, Reconstructive and Hand Surgery, Amsterdam UMC, Amsterdam Movement Sciences, Vrije Univeristeit Amsterdam, 1081 HV Amsterdam, The Netherlands; 3 Burn Centre, Red Cross Hospital, 1942 LE Beverwijk, The Netherlands; 4 Department of Traumasurgery, Leiden University Medical Centre, 2333 ZA Leiden, The Netherlands; 5 Department of Surgery, Amsterdam Movement Sciences, Amsterdam UMC, Vrije Univeristeit Amsterdam, 1081 HV Amsterdam, The Netherlands; 6 Association of Dutch Burn Centres, Red Cross Hospital, 1942 LE Beverwijk, The Netherlands; 7 Pediatric Surgical Centre, Emma Children’s Hospital, Amsterdam UMC, University of Amsterdam, Vrije Universiteit, 1105 AZ Amsterdam, The Netherlands; 8 Burn Centre, Martini Hospital, 9728 NT Groningen, The Netherlands; 9 Association of Dutch Burn Centres, Martini Hospital, 9728 NT Groningen, The Netherlands; 10 Research Group Healthy Ageing, Allied Health Care and Nursing, Hanze University of Applied Sciences Groningen, 9747 AS Groningen, The Netherlands; 11 Department for Human Movement Sciences, University Medical Center Groningen, 9713 GZ Groningen, The Netherlands; 12 Department of Public Health, Erasmus MC, University Medical Centre Rotterdam, 3015 GD Rotterdam, The Netherlands; 13 Association of Dutch Burn Centres, Maasstad Hospital, 3079 DZ Rotterdam, The Netherlands; 14 Trauma Research Unit, Department of Surgery, Erasmus MC, University Medical Centre Rotterdam, 3015 GD Rotterdam, The Netherlands

## Abstract

**Background:**

Tangential excision of burned tissue followed by skin grafting is the cornerstone of burn surgery. Hydrosurgery has become popular for tangential excision, with the hypothesis that enhanced preservation of vital dermal tissue reduces scarring. The aim of this trial was to compare scar quality after hydrosurgical *versus* conventional debridement before split-skin grafting.

**Methods:**

A double-blind randomized within-patient multicentre controlled trial was conducted in patients with burns that required split-skin grafting. One wound area was randomized to hydrosurgical debridement and the other to Weck knife debridement. The primary outcome was scar quality at 12 months, assessed with the observer part of the Patient and Observer Scar Assessment Scale (POSAS). Secondary outcomes included complications, scar quality, colour, pliability, and histological dermal preservation.

**Results:**

Some 137 patients were randomized. At 12 months, scars of the hydrosurgical debrided wounds had a lower POSAS observer total item score (mean 2.42 (95 per cent c.i. 2.26 to 2.59) *versus* 2.54 (95 per cent c.i. 2.36 to 2.72; *P* = 0.023)) and overall opinion score (mean 3.08 (95 per cent c.i. 2.88 to 3.28) *versus* 3.30 (95 per cent c.i. 3.09–3.51); *P* = 0.006). Patient-reported scar quality and pliability measurements were significantly better for the hydrosurgically debrided wounds. Complication rates did not differ between both treatments. Histologically, significantly more dermis was preserved with hydrosurgery (*P* < 0.001).

**Conclusion:**

One year after surgery scar quality and pliability was better for hydrosurgically debrided burns, probably owing to enhanced histological preservation of dermis.

**Registration number:**

Trial NL6085 (NTR6232 (http://www.trialregister.nl)).

## Teaser

In this randomized controlled trial, scar quality and pliability were better for burns debrided with hydrosurgery 1 year after skin grafting This was probably the result of enhanced dermal preservation, as assessed by histology.

## Introduction

Early debridement and split-skin grafting is the standard of care for deep dermal burn wounds to maximize recovery of the affected area and minimize pathological scarring^1^.

Conventional surgical debridement consists of sharp tangential excision of non-viable tissue with hand-held knives until bleeding tissue is encountered (a marker of vital tissue)^2^. Commonly used instruments for conventional debridement include the Watson knife, the Humby knife, the Goulian, or the Weck knife. Hydrosurgical debridement is an alternative to conventional knife debridement. The principle of hydrosurgery is the emission of a jet of water across an aperture that causes a localized vacuum to cut, irrigate, and suction tissue simultaneously. The speed of the water jet can be adjusted by the surgeon and is claimed to lift only non-viable tissue, thereby achieving accurate wound debridement with maximal preservation of viable dermis^3^.

Loss of dermis has been considered as one of the main factors determining the quality of a scar^4^. Burn specialists widely use hydrosurgery as an alternative for conventional tangential debridement. The underlying hypothesis is that scar quality will be better after hydrosurgical debridement as it enables surgeons to debride burned tissue accurately, with maximal preservation of viable dermis, in contrast to conventional surgical debridement, which is associated with unnecessary tissue loss. A recent Cochrane review showed uncertainty over whether or not hydrosurgical debridement and skin grafting is better than conventional surgical debridement and skin grafting for the treatment of acute partial-thickness burns and concluded that more trials are needed^5^.

The aim of this study was to compare and evaluate the long-term scar quality of patients whose burns were debrided with hydrosurgical or conventional techniques before split-skin grafting.

## Methods

The HyCon study (long-term scar quality after hydrosurgical *versus* conventional debridement for deep dermal burns) is a multicentre, within-patient randomized, double-blind controlled trial. The Medical Ethics Committee and Institutional Review Boards of each participating hospital approved the study protocol, which has been published elsewhere^6^. The last version of the study protocol is available in *Appendix S1*. The study was registered with the Netherlands Trial Register before the start of recruitment (Trial NL6085 (NTR6232)). The study was conducted according to the principles of the Declaration of Helsinki (World Medical Association Revision 2013), the Medical Research Involving Human Subjects Act (WMO), and the CONSORT statement for reporting within-person randomized trials^7^.

### Setting and recruitment

Participants were recruited in the three specialized burn centres in the Netherlands (Maasstad Hospital in Rotterdam; the Martini Hospital in Groningen; and the Red Cross Hospital in Beverwijk). National guidelines advise referral to one of these specialized burn centres if a patient fulfils one of the Emergency Managements of Severe Burns referral criteria^8,9^.

Eligible patients had burns with a surface area larger than 50 cm^2^ that required debridement and split-skin grafting. There was no age restriction. Patients with full-thickness burns were excluded as the hydrosurgery system cannot cut through hard, leather-like eschar. Other exclusion criteria were wound infection, insufficient knowledge of the Dutch or English language, and patients who were unlikely to comply with the requirements of the follow-up. Patients or their legal representatives gave written informed consent before being included in the study. The inclusion criteria were adapted to overcome low eligibility rates in the first months of the inclusion period. Contrary to the published protocol, both study areas did not have to be adjacent if they were both suitable for hydrosurgical and conventional debridement and of equal depth, as determined by an experienced burn physician (preferably in combination with a laser Doppler imaging scan). Patients with a total body surface area (TBSA) burned of more than 30 per cent were included^6^.

### Procedures and interventions

Every participant acted as their own control. Two similar wound areas (assigned A or B) of at least 25 cm^2^ were selected in each patient by the surgeon. If possible, the study areas were adjacent. Otherwise, a similar burn wound at the contralateral body part or the nearest comparable burn wound was chosen. Photographs were taken to facilitate identification of both areas during follow-up. After assignment by the surgeon and before surgery started, the study areas were randomly allocated, using a web-based automated randomization system (https://data.castoredc.com), to either hydrosurgical or conventional debridement with a Weck knife in the operating room by a member of the research team. During the operation, the study areas were debrided with the VERSAJET™ Hydrosurgery System (Smith + Nephew, London UK) or conventional surgery using a hand-held knife. Both study areas were debrided during the same procedure and covered with the same size of meshed split-skin graft or Meek wall grafts and identical non-adhesive wound dressings. Graft harvesting, meshing, and fixation were done following local treatment protocols. This design allowed comparison of hydrosurgical *versus* conventional debridement within the same participant while controlling for variations in healing and scarring that could occur between patient groups. Dressings were left *in situ* for 5–7 days. Both study areas were followed until complete wound closure (at least 95 per cent re-epithelialization) was achieved, assessed by a member of the research team and documented with photographs and notes from a clinician in the patient’s file as instructed by the standard operating procedure. Standard of care involved visits to the outpatient clinic at 3, 6, and 12 months after surgery. Clinicians/researchers who assessed scar quality during follow-up and patients were blinded to the modality used to debride the study areas.

### Baseline characteristics

Investigators not involved in the clinical care of participants were responsible for trial recruitment, allocation, and data collection. They recorded the following baseline characteristics for all included patients: age, sex, Fitzpatrick skin type, comorbidities, percentage TBSA burned, wound aetiology, burn depth, and time to surgery. During surgery, Weck knife, Versajet settings, and skin graft expansion were recorded.

### Primary outcome measure

The primary outcome was scar quality at 12 months, assessed by the clinician/researcher with the Patient and Observer Scar Assessment Scale (POSAS) version 2.0^10^. The POSAS questionnaire consists of two six-item scales; one for the observer (clinician/researcher) and one for the patient. The observer total item score was chosen as the primary outcome because it has been demonstrated to produce valid and reproducible results by trained evaluators^11,12^. The observer part includes the items ‘vascularity’, ‘pigmentation’, ‘thickness’, ‘relief’, ‘pliability’, and ‘surface area’. These items were separately scored on a 10-point rating scale with a score of 1 corresponding to the situation of normal skin and 10 indicating the worst imaginable scar. Two independent observers scored the scar quality, to improve the reliability of the assessment^11^. The mean score of the items of both observers formed the observer total item score^11,12^.

### Secondary outcome measures

Secondary outcome measures included complications and wound healing, scar colour, scar pliability, and dermal preservation measured by histology.

#### Complications and wound healing

The presence of complications were registered per study area. Infection was defined by clinical signs in combination with a positive swab. Graft loss was registered in percentages to measure small losses. Prolonged wound healing was defined as taking more than 2 weeks to achieve 95% or more re-epithelialization.

#### Scar quality measures

Patient-reported scar quality was measured with the total item score of the patient part of the POSAS. The patient part of the POSAS includes the parameters pain, itch, colour, thickness, relief, and pliability. Pain and itch were scored between 1 (no pain/itch) and 10 (extreme pain/itch). Each of the other items was scored between 1 (no difference with normal skin) and 10 (very different from normal skin). The mean score of these items formed the patient total item score. In addition to the item scores, both observers and patients gave a score for their overall opinion on the scar on a 10-point rating scale, where 1 resembles normal skin and 10 resembles the worst imaginable scar. Because scar quality changes over time, the POSAS was used to assess scar quality during standard follow-up visits to the outpatient clinic at 3, 6, and 12 months postoperatively. At the 12-month visit, patients were asked to indicate the degree of clinical difference they noticed between study area A and B on a 5-point Likert scale (much worse, worse, the same, better, a lot better). The purpose of this question was to gain insight into patients’ perspectives regarding what is clinically important.

#### Scar colour

Scar colour was evaluated measuring erythema and melanin with the DSM II ColorMeter (Cortex Technology, Hadsund, Denmark). Colour results were expressed as the absolute difference between healthy skin and scar to eliminate the season-related influence of sun exposure on the erythema and melanin scores.

#### Scar pliability

Scar pliability was measured using the Cutometer Skin Elasticity Meter 575 (Courage and Khazaka, Cologne, Germany). The Cutometer measures the vertical deformation of the skin in millimetres into the circular aperture of the probe after a controlled vacuum. Two Cutometer parameters previously shown to be the most reliable were used: elasticity (Ue) and maximal extension (Uf)^13^. To eliminate the influence of different anatomical locations, elasticity was analysed using the ratio of the scar to normal skin.

#### Colour and pliability measurement procedures

To prevent measurement bias within the scar, the colour and pliability measurements were performed on five locations, following a standardized method that includes five scar measurements^14^. The average score of these five scar measurements was used. The first option for the control measurement was the patient’s unaffected contralateral site. In cases where the contralateral site was also affected, the most comparable and unaffected spot near the scar was used. Measurements were performed at 3 and 12 months postsurgery.

#### Dermal preservation

The amount of remaining dermis after debridement was evaluated by histopathology. During surgery, punch biopsies (diameter 3 mm) were taken from both study areas after debridement. In addition to the haematoxylin and eosin staining described in the previously published protocol, we used Herovici polychrome staining to analyse the biopsies and to differentiate between mature collagen and granulation tissue^15,16^. All resection specimens were processed and sampled using a standard protocol (*Appendix S3*)

### Statistical analysis

A sample size calculation was performed based on the results of an unpublished retrospective study on scar quality after hydrosurgery *versus* guarded knife excision, assessed using photographs taken by carers^17^. In this study, the total item score of the observer scale of the POSAS questionnaire 12 months postsurgery was 14.7 in the hydrosurgery group and 16.7 in the conventional debridement group, with a pooled standard deviation (s.d.) of 6.53, resulting in an effect size of 0.3. Given a power of 0.90, a significance level of 0.05, and including a correction for correlated samples and those lost to follow-up, a sample size of 137 was calculated. Continuous data were first tested for normality. Normally distributed data are presented as mean (95 per cent confidence interval (c.i.)) and testing was performed with paired *t* tests. Non-normally distributed data are presented as median (interquartile range (i.q.r.)) values and analysed with the Wilcoxon signed ranks test for paired data. Effect sizes for the paired *t* test were represented using Cohen’s d; for Wilcoxon signed rank test, *r* was used^18^. The McNemar test was used for paired dichotomous values and odds ratios were used to represent the effect size. Because of repeated measurements within patients, overall differences between both treatments in subjective scar-quality outcomes was analysed using generalized estimating equation (GEE) with an exchangeable correlation matrix structure. Significance was set at *P* < 0.05. Analyses were conducted using SPSS^®^ version 25 (IBM, Armonk, New York, USA) and Stata^®^ version 16 (StataCorp, College Station, Texas, USA).

## Results

**Fig. 1:**
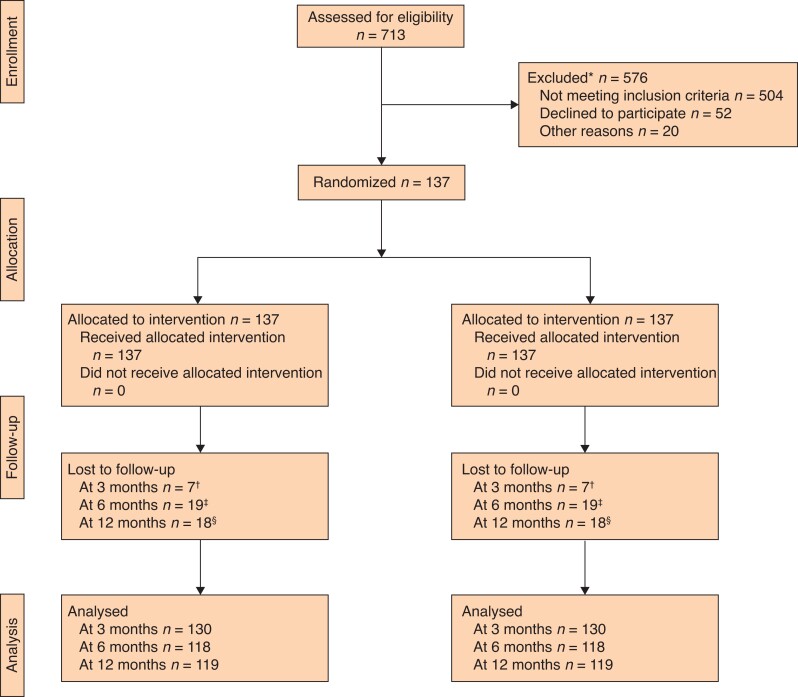
CONSORT diagram for the trial

**Fig. 2:**
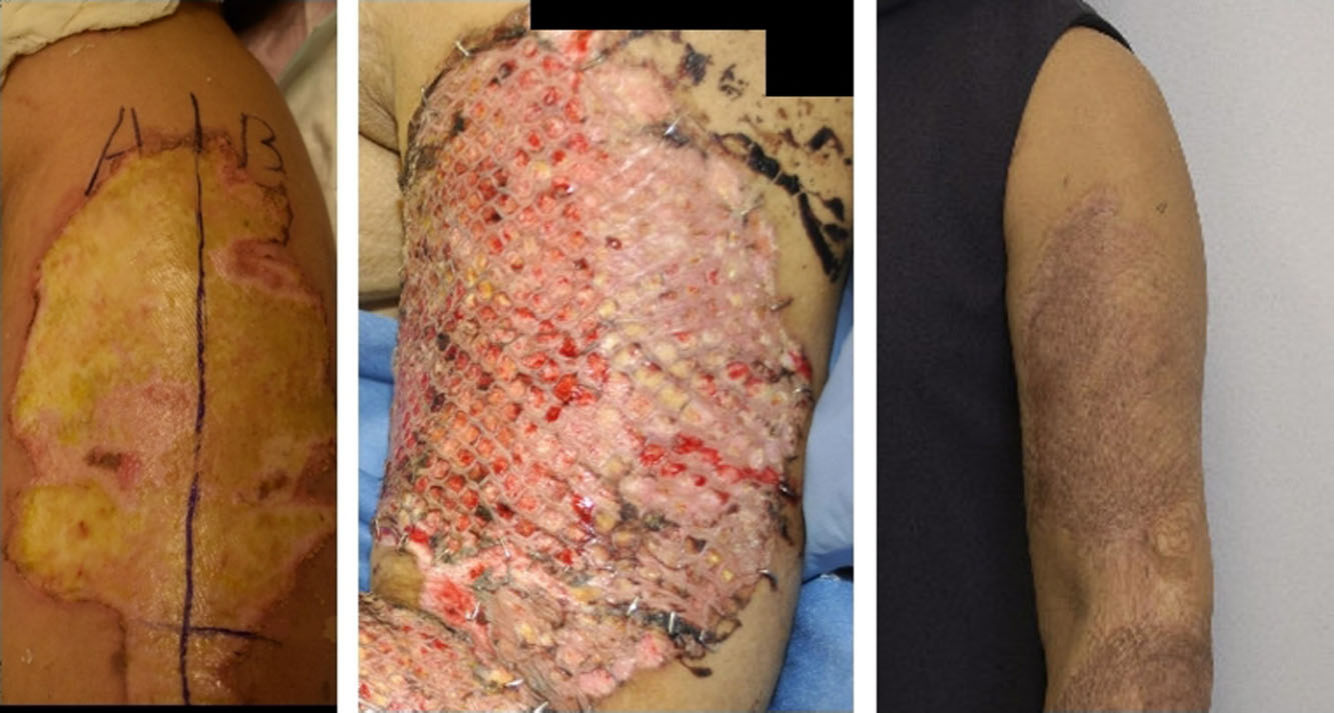
Allocation of wound areas on the left upper arm before randomization (left), wound inspection 5 days after surgery (middle), and scarring at 12 months of follow-up (right) in a 61-year-old female

From January 2017 to July 2019, 713 patients were screened for inclusion, of whom 137 were eligible to be randomized (*Figs 1 and 2* and *Table 1*).

*‘Other’ reasons for exclusion were *missed by the study team* or *participation in another intervention study.*^†^Six lost to follow-up, one dropout because of new self-inflicted burn wounds in both study areas. ^‡^Seventeen lost to follow-up, on dropout because of new wounds in study areas, one deceased. ^§^Sixteen lost to follow-up, one dropout because of new wounds on study areas, and one deceased. Specification of the number of patients per exclusion criterion is available online (*Appendix S2*).

The part marked ‘A’ was randomized to debridement with a Weck knife and part B was randomized to debridement with hydrosurgery.

**Table 1:** Baseline characteristics

Characteristic	Total (*n* = 137)
Age (years)*	45 (25–59)
**Sex ratio (F:M)**	54:83
**Skin type**	
Fitzpatrick 1–2	79 (57)
Fitzpatrick 3–4	49 (36)
Fitzpatrick 5–6	9 (7)
**Diabetes**	11 (8)
% TBSA burned*	7 (4–14)
**Aetiology**	
Flame	85 (62)
Scald	26 (19)
Fat	20 (15)
Other	6 (4)
**Surgical characteristics**	
Time from injury to surgery (days)*	15 (10–19)
Versajet setting^†^	5.25 (4.82–5.69)
Blade Weck knife	
0.008 inch	17 (12)
0.010 inch	19 (14)
0.012	81 (59)
Unknown	20 (15)
Skin graft expansion	
1:1	16 (12)
1:1.5	66 (48)
1:2	10 (7)
1:3	30 (22)
1:6 (Meek technique)	15 (11)

Values in parentheses are percentages unless indicated otherwise. *Median (i.q.r.). †Mean (s.d.). TBSA, total body surface area.

**Table 2:** Patient and Observer Scar Assessment Scale (POSAS) scores 12 months postsurgery

	Hydrosurgical debridement	Conventional debridement	Effect size*	*P*- value
**Observer scar score**				
Total item score^†^	2.42 (2.26–2.59)	2.54 (2.36–2.72)	−0.21	0.023^‡^
Vascularity^†^	2.47 (2.26–2.69)	2.59 (2.37–2.81)		
Pigmentation^†^	2.81 (2.62–3.00)	2.89 (2.70–3.08)		
Thickness^†^	2.30 (2.08–2.51)	2.81 (2.57–3.19)		
Relief^†^	2.66 (2.44–2.87)	2.81 (2.58–3.05)		
Pliability^§^	2.50 (1.50–3.00)	2.50 (1.50–3.50)		
Surface area^§^	1.50 (1.00–2.00)	1.50 (1.00–2.00)		
Overall opinion score^†^	3.08 (2.88–3.28)	3.30 (3.09–3.51)	−0.25	0.006^‡^
**Patient scar score**				
Total item score^§^	2.68 (1.67–4.33)	3.00 (1.83–4.83)	−0.14	0.019^¶^
Pain^§^	1 (1–1)	1 (1–1)		
Pruritus^§^	1 (1–3)	1 (1–3)		
Colour^§^	4 (3–6)	4 (3–6)		
Stiffness^§^	3 (1–5)	3 (1–6)		
Thickness^§^	2 (1–5)	3 (1–6)		
Relief^§^	3 (1–5)	4 (2–6)		
Overall opinion score^§^	4 (2–6)	4 (3–6)	−0.15	0.024^¶^

POSAS scores range from 1 to 10. A lower score correlates with a better scar. Observer scores are the mean scores of the six items scored by two clinicians/researchers. *Effect size for paired t test represented using Cohen’s d. Effect size for Wilcoxon signed rank test represented using Cohen’s r. †Mean (95 per cent c.i.). ‡Paired t test. §Median (i.q.r.). ¶Wilcoxon signed rank test.

### Primary outcome

Scar quality measured with the observer total item score of the POSAS at 12 months was better for hydrosurgically debrided burns than for the conventional debrided burns (mean difference −0.12 (95 per cent c.i. −0.22 to −0.02); *P* = 0.023)) (*Table 2*).

### Secondary outcomes

#### Wound healing and complications

Time to re-epithelialization did not differ between both intervention groups. No significant differences in wound infection, percentage graft loss, and other complications were found between treatment groups (*Table 3*).

**Table 3:** Secondary outcomes

	Hydrosurgical debridement (*n* = 137)	Conventional debridement (*n* = 137)	Hydrosurgical *versus* conventional	*P* value*
			Odds ratio	
**Complications**				
Wound infection	19 (13.9)	20 (14.6)	0.93 (0.47–1.86)	1.000^†^
Graft loss (partial or total)	3 (2.2)	8 (5.8)	0.36 (0.09–1.39)	0.227^†^
Prolonged wound healing	29 (21.1)	22 (16.1)	1.40 (0.68–2.87)	0.065^†^
Other	1 (0.7)	3 (2.2)	0.33 (0.03–3.22)	0.625^†^
			Effect size^‡^	
**Wound healing**				
Re-epithelialization 5–7 days postsurgery (%)^§^	80.0 (68.96–79.64)	81.2 (73.20–82.32)	−0.13	0.144
Time to re-epithelialization (days)^¶^	7 (5–13)	7 (5–12)	−0.10	0.353^#^
**Observer POSAS sores**				
Total item score				
3 months^§^	3.04 (2.91–3.22)	3.18 (3.03–3.36)	−0.21	0.021
6 months^§^	2.72 (2.57–2.91)	2.93 (2.75–3.10)	−0.29	0.002
Overall opinion score				
3 months^§^	3.87 (3.69–4.11)	4.07 (3.86–4.27)	−0.21	0.031
6 months^§^	3.51 (3.33–3.74)	3.73 (3.54–3.94)	−0.26	0.006
**Patient POSAS sores**				
Total item score				
3 months^§^	4.14 (3.79–4.50)	4.55 (4.22–4.88)	−0.28	0.002
6 months^¶^	3.33 (2.21–4.83)	3.67 (2.54–5.17)	−0.11	0.093^#^
**Overall opinion score**				
3 months^¶^	5 (3–7)	6 (4–8)	−0.14	0.011^#^
6 months^¶^	5 (2–6)	5 (3–7)	−0.21	0.001^#^
Colour**				
Erythema				
3 months^¶^	6.26 (3.38–10.67)	6.68 (3.11–10.35)	−0.02	0.560^#^
12 months^¶^	3.61 (2.08–8.82)	4.08 (1.60–9.51)	−0.04	0.596^#^
Melanin				
3 months^¶^	13.24 (6.97–21.72)	13.96 (8.13–22.54)	−0.16	0.041^#^
12 months^¶^	7.19 (4.30–13.87)	6.24 (3.77–15.02)	0.24	0.607^#^
**Pliability^††^**				
Elasticity (Ue)				
3 months^¶^	0.55 (0.35–0.74)	0.49 (0.32–0.71)	0.08	0.225^#^
12 months^¶^	0.73 (0.58–0.91)	0.70 (0.53–0.89)	0.15	0.029^#^
Maximal extension (Uf)				
3 months^§^	0.58 (0.50–0.72)	0.55 (0.47–0.62)	0.18	0.089
12 months^¶^	0.75 (0.62–0.91)	0.72 (0.56–0.90)	0.14	0.039^#^
Histopathological findings				
Dermal preservation (µm)^¶^	1748 (1213–2175)	1265 (689–1989)	0.23	<0.001^#^

Values in parentheses are percentages unless indicated otherwise. *Paired t test unless indicated otherwise. †McNemar’s test. ‡Effect size for paired t test represented using Cohen’s d. Effect size for Wilcoxon signed rank test represented using Cohen’s r^18^. §Mean (95 per cent c.i.). ¶Median (i.q.r.). #Wilcoxon signed rank test. **Means are calculated as absolute difference between scar tissue and the non-affected skin. ††Values represent the ratio between scar tissue and non-affected skin.

#### Scar quality

One year after surgery, scar quality scores were significantly lower (i.e. reflecting a better scar) for hydrosurgically debrided burns in terms of the observer overall opinion score, the patient total item score, and patient overall opinion score (*Table 2*). Observer and patient reported POSAS scores at 3 and 6 months postsurgery are provided in *Table 3.* There was a better outcome for the hydrosurgically debrided wounds over time in terms of the observer total item score (mean difference −0.16 (95 per cent c.i. −0.25 to −0.06; *P* = 0.001)), observer overall opinion score (mean difference −0.22 (95 per c.i. −0.34 to −0.09, *P* = 0.001)), and patient total item score (mean difference −0.29 (95 per cent c.i. −0.49 to −0.09; *P* = 0.0024)) but not for the patient overall opinion score (mean difference −0.18 (95 per cent c.i. −0.75 to 0.40; *P* = 0.547)) using GEE analyses. At 12 months postsurgery, 56 patients (48 per cent) rated the hydrosurgically debrided study area as a better scar on the Likert scale, 30 patients (26 per cent) rated the conventionally debrided study area as better, and 30 patients (26 per cent) said they noticed no difference between both study areas.

#### Scar colour

The erythema index of the hydrosurgically debridement and conventionally debrided study areas did not differ significantly at 3 and 12 months postsurgery (*Table 3*). The melanin index for scars of wounds that were treated with hydrosurgery were significantly more comparable to normal skin at 3 months but did not differ at 12 months (*Table 3*).

#### Scar pliability

At 12 months postsurgery, the scars of hydrosurgically debrided wounds were more comparable to normal skin in terms of the pliability parameters of elasticity and maximal extension (*P* = 0.029 and *P* = 0.039, respectively; *Table 3*).

#### Histopathological findings

Of the hydrosurgically debrided study areas, 104 biopsies were included for analyses. Of the conventionally debrided study areas, 101 biopsies qualified for analyses. More dermis was left in the punch biopsies of wounds that were debrided with hydrosurgery (*P* < 0.001; *Table 3*).

## Discussion

The use of hydrosurgery led to better scar-quality outcomes, as reported by clinicians and patients, up to 1 year postsurgery. Objective scar pliability measures were also significantly better, which was probably the result of better preservation of dermis after hydrosurgical debridement.

An important topic to discuss is whether statistical differences in POSAS outcomes present a clinically significant difference. Effect sizes in observer outcomes were small (ranging from −0.21 to −0.29) but not trivial^19^. However, the effect sizes of patient outcomes were smaller (ranging from −0.11 to −0.28)^20^. Of the trial population, 48 per cent considered the hydrosurgically debrided study area as better or much better at 12 months after surgery *versus* 26 per cent of the conventionally debrided study area. In addition, unpublished data from our institute suggest that patients consider differences between −0.08 and −0.39 in patient POSAS item scores as important, which may indicate that the differences identified are at least of some importance to patients. However, in the absence of a minimal clinically important difference in POSAS score, uncertainty remains over what difference in outcome should be considered clinically important.

The goal of debridement of a burn wound is to remove injured and non-viable tissue to create the optimal wound bed for autologous split-thickness skin grafting^21–23^. An essential asset of an effective debridement tool is to remove as much necrotic tissue as possible while preserving as much vital tissue as possible, to improve clinical, functional, and cosmetic outcomes^4,24,25^. Although specialists in burns have long recognized the association between the depth of dermal injury and the degree of scarring, the cellular and molecular basis of the relationship remains poorly understood. Dunkin *et al*. hypothesized that different depths of dermal injury may result in different inflammatory responses and cytokine profiles, which, in turn, provide an environment for a different proliferative response^25^. The current study is the first to report a relationship between dermal preservation and better clinical scar outcomes. POSAS item scores related to elasticity (thickness, relief, pliability, and stiffness) differed most between both study groups. These results, in combination with better measurement of pliability for scars after hydrosurgical debridement, suggest that the preservation of dermis leads to better scar quality in terms of how scars ‘feel’ rather than how scars ‘look’ (vascularity, pigmentation, and colour). Further studies are necessary to better understand the relationship between dermal preservation (i.e. selective debridement) and scar quality. Time to wound healing and complication rates did not differ between both treatment groups and can therefore be excluded as causes for superior scar quality after hydrosurgical debridement. This also implies that both techniques provided sufficient debridement.

Although several studies have reported that hydrosurgery can be used maximally to preserve dermis, only one has reported histological evidence to support this in burns^26–31^. Hyland *et al*. performed an RCT to study scar quality after hydrosurgical and conventional debridement in children with partial-thickness burns^30^. They also confirmed greater loss of dermis in conventionally debrided burns via an analysis of histological specimens. They also found better scar scores in favour of the hydrosurgery group at 3 and 6 months postburn, but this difference was not statistically significant. However, they did not use a within-patient design, did not report the distribution of patient characteristics that may have influenced scar quality, and ended their follow-up at 6 months.

A recent Cochrane review reported low-quality evidence for the potential benefits of hydrosurgical debridement over conventional debridement that are desirable to clinicians, such as faster operating time, improved usability, fewer procedures, less blood loss, and a shorter hospital stay^5^. To reduce treatment costs, burn specialists often prefer to use a Weck knife in smaller burns. The current cost of the disposable Versajet headpiece is €141.86 ($167.55), while the costs of a reusable Weck knife guard and handle are €0.91 ($1.08) and €20.91 ($24.70), respectively. The cost of one sterilized, single-use Weck blade is €1.08 ($1.28). However, evidence for the cost-effectiveness of hydrosurgery in burns is limited and further research on its long-term benefits, such as fewer reconstructive surgery procedures, is necessary^32^.

The strengths of this trial include the comprehensive inclusion criteria (patients of all ages and most burn aetiologies), which allowed application to a broad patient population. The within-patient design reduced factors that could lead to confounding of scar outcomes. Much effort was put into minimizing the risk of bias due to incomplete outcome data, which resulted in a remarkably low drop-out rate after 1 year follow-up (13 per cent). The multicentre approach and pragmatic character (mimicking routine clinical practice) improve the generalizability of the results. Another strength is the use of the POSAS instrument; it is validated, incudes most relevant scar characteristics, and is the most frequently used scale^33–36^.

The key assets of the trial also create the main limitations, including the within-patient design and outcome measure. For patients, the POSAS may have been difficult to rate for two study areas. In particular, after adjustment of the protocol that allowed study areas to be on different parts of the body, the patient’s opinion on scar quality might have been biased based on the location of the scar, especially if scars are further apart on the body or if one is in the sight and the other is not. To increase reliability, the observer part of the POSAS was used as the primary outcome, and was scored by two independent trained observers. To improve the feasibility of the trial, there were no restrictions or standards for delivery of the intervention, depth of graft harvesting, skin graft fixation, or wound treatment after surgery. However, both wound areas were treated the same within patients and therefore this may lead to improvement of generalizability of the results rather than bias of scar-quality outcomes. Previous research has shown that burn surgeons tend to use hydrosurgery more often in children and more superficial wounds (like scalds)^6^. Subgroup analyses were not part of the initial research plan and further research is necessary to gain insight into the benefits of hydrosurgery for different patient and burn categories. Scar outcomes present data from specialized burn care, which might not be comparable to outcomes in non-specialized centres. Nevertheless, hydrosurgical debridement is easy to learn and the device is not difficult to use. Therefore, its use may lead to better scar outcomes in centres where surgeons are not frequently practising burn debridement with guarded knives.

## Collaborators

Collaborators of the HyCon study group: J. Dokter; K. L. M. Gardien; J. Hiddingh, H. W. C. Hofland; Y. Lucas; A. Meij-de Vries; T. H. J. Nijhuis; I. M. M. H. Oen; D. T. Roodbergen; S. M. H. J. Scholten-Jaegers; M. Stoop; M. Vlig; N. Trommel.

## Funding

This research was funded by the Dutch Burns Foundation (grant number 15.101).

*Disclosure.* The authors declare no conflict of interest.

## Supplementary material

Supplementary data are available at *BJS* online.

## Supplementary Material

znab470_Supplementary_Data
