# Supplementary material for: Hydrosurgical and conventional debridement of burns: randomized clinical trial
Source: Br J Surg. 2022 Mar 3;109(4):332–9. doi: 10.1093/bjs/znab470 (PMC10364696; doi:10.1093/bjs/znab470)
Supplement: znab470_Supplementary_Data [file znab470_supplementary_data.zip › Supplementary_Appendix_1.docx]

**RESEARCH PROTOCOL**

**A multicentre randomized controlled trial comparing long term scar quality after hydrosurgical versus conventional tangential excision of deep dermal burns**

Version 4.2, June 2017

**PROTOCOL TITLE** ‘A multicentre randomized controlled trial comparing long term scar quality after hydrosurgical versus conventional tangential excision of deep dermal burns’

| Protocol ID | NL58875.101.16 |
| --- | --- |
| Short title | *HyCon trial* |
| EudraCT number | *Not applicable* |
| Version | *4.2* |
| Date | *June 2017* |
| Coordinating investigator | *C.M. Legemate, MD*  *Maasstad Hospital*  *Postbus 9100*  *3007 AC Rotterdam*  *e-mail:* [*legematec@maasstadziekenhuis.nl*](mailto:legematec@maasstadziekenhuis.nl) |
| *Principal investigator(s)* | ***Rotterdam:***  *C.H. van der Vlies, MD, PhD*  *Maasstad Hospital*  *Postbus 9100*  *3007 AC Rotterdam*  *Email: vliesc@maasstadziekenhuis.nl*  ***Beverwijk:***  *P.P.M. van Zuijlen, MD, PhD*  *Red Cross Hospital*  *Postbus 1074*  *1940 EB Beverwijk*  *Email:* [*pvzuijlen@rkz.nl*](mailto:pvzuijlen@rkz.nl)  ***Groningen:***  *M.K. Nieuwenhuis, PhD*  *Martini Hospital*  *Postbus 30.033*  *9700 RM Groningen*  *Email: m.k.nieuwenhuis@mzh.nl* |
| Sponsor | *Prof. dr. E. Middelkoop*  *Association of Dutch Burn Centres (ADBC)*  *Postbus 1015*  *1940 EA Beverwijk* |
| Subsidising party | *Dutch Burn Foundation*  *Grant number: WO/15.101* |
| Independent expert (s) | *M.G.A. Baartmans, MD*  *Maasstad Hospital*  *Postbus 9100 3007 AC Rotterdam*  *Email:* [*baartmansm@maasstadziekenhuis.nl*](mailto:baartmansm@maasstadziekenhuis.nl) |

**PROTOCOL SIGNATURE SHEET**

| Name | Signature | Date |
| --- | --- | --- |
| **Head of Department:**  *Prof. dr. E. Middelkoop*  *Research Director ADBC* |  |  |
| **Project leader:**  *C.H. van der Vlies, MD, PhD Trauma and burn surgeon Maasstad Hospital Rotterdam, the Netherlands* |  |  |

**TABLE OF CONTENTS**

1. INTRODUCTION AND RATIONALE 10

2. OBJECTIVES 13

3. STUDY DESIGN 14

4. STUDY POPULATION 15

4.1 Population (base) 15

4.2 Inclusion criteria 15

4.3 Exclusion criteria 15

4.4 Sample size calculation 15

5. TREATMENT OF SUBJECTS 16

5.1 Investigational product/treatment 16

5.2 Use of co-intervention (if applicable) 16

5.3 Escape medication (if applicable) 16

6. INVESTIGATIONAL PRODUCT 17

6.1 Name and description of investigational product(s) 17

6.2 Summary of findings from non-clinical studies 17

6.3 Summary of findings from clinical studies 18

6.4 Summary of known and potential risks and benefits 20

6.5 Description and justification of route of administration and dosage 20

6.6 Dosages, dosage modifications and method of administration 20

6.7 Preparation and labelling of Investigational Medicinal Product 21

6.8 Drug accountability 21

7. NON-INVESTIGATIONAL PRODUCT 22

8. METHODS 23

8.1 Study parameters/endpoints 23

8.1.1 Main study parameter/endpoint 23

8.1.2 Secondary study parameters/endpoints (if applicable) 23

8.1.3 Other study parameters (if applicable) 23

8.2 Randomisation, blinding and treatment allocation 23

8.3 Study procedures 24

8.4 Withdrawal of individual subjects 27

8.4.1 Specific criteria for withdrawal (if applicable) 28

8.5 Replacement of individual subjects after withdrawal 28

8.6 Follow-up of subjects withdrawn from treatment 28

8.7 Premature termination of the study 28

9. SAFETY REPORTING 29

9.1 Temporary halt for reasons of subject safety 29

9.2 AEs, SAEs and SUSARs 29

9.2.1 Adverse events (AEs) 29

9.2.2 Serious adverse events (SAEs) 29

9.2.3 Suspected unexpected serious adverse reactions (SUSARs) 29

9.3 Annual safety report 30

9.4 Follow-up of adverse events 31

9.5 Data Safety Monitoring Board (DSMB) / Safety Committee 31

10. STATISTICAL ANALYSIS 32

10.1 Primary study parameter(s) 32

10.2 Secondary study parameter(s) 32

10.3 Other study parameters 32

10.4 Interim analysis (if applicable) 32

11. ETHICAL CONSIDERATIONS 33

11.1 Regulation statement 33

11.2 Recruitment and consent 33

11.3 Objection by minors or incapacitated subjects (if applicable) 33

11.4 Benefits and risks assessment, group relatedness 33

11.5 Compensation for injury 33

11.6 Incentives (if applicable) 34

12. ADMINISTRATIVE ASPECTS, MONITORING AND PUBLICATION 35

12.1 Handling and storage of data and documents 35

12.2 Monitoring and Quality Assurance 35

12.3 Amendments 35

12.4 Annual progress report 36

12.5 Temporary halt and (prematurely) end of study report 36

12.6 Public disclosure and publication policy 36

13. STRUCTURED RISK ANALYSIS 37

13.1 Potential issues of concern 37

13.2 Synthesis 37

14. REFERENCES 38

**LIST OF ABBREVIATIONS AND RELEVANT DEFINITIONS**

| **AE** | **Adverse Event** |
| --- | --- |
| **DSMB** | **Data Safety Monitoring Board** |
| **EU** | **European Union** |
| **GCP** | **Good Clinical Practice** |
| **IC** | **Informed Consent** |
| **METC** | **Medical research ethics committee (MREC); in Dutch: medisch ethische toetsing commissie (METC)** |
| **MIC** | **Minimal Clinical Important Change** |
| **MCID** | Minimal Clinically Important Difference |
| **POSAS** | **Patient and Observer Scar Assessment Scale** |
| **RCT** | **Randomized controlled trial** |
| **(S)AE** | **(Serious) Adverse Event** |
| **SPC** | **Summary of Product Characteristics (in Dutch: officiële productinfomatie IB1-tekst)** |
| **SPSS** | **Statistical Package for the Social Sciences** |
| **Sponsor** | **The sponsor is the party that commissions the organisation or performance of the research, for example a pharmaceutical**  **company, academic hospital, scientific organisation or investigator. A party that provides funding for a study but does not commission it is not regarded as the sponsor, but referred to as a subsidising party.** |
| **SUSAR** | **Suspected Unexpected Serious Adverse Reaction** |
| **TBSA** | **Total Body Surface Area** |
| **WMO** | **Medical Research Involving Human Subjects Act (in Dutch: Wet Medisch-wetenschappelijk Onderzoek met Mensen** |
|  |  |
|  |  |
|  |  |
|  |  |
|  |  |
|  |  |
|  |  |
|  |  |
|  |  |
|  |  |
|  |  |

**SUMMARY**

**Rationale:** Surgical debridement is an important step in the management of patients with deep dermal or full thickness burns. Its purpose is to remove necrotic and/or infectious tissue and to prepare the wound for definitive healing. Conventional surgical debridement of burn wounds prior to skin grafting commonly consists of sharp tangential excision of non-viable burn eschar with hand-held knives. This procedure is not only associated with substantial blood loss, but also with unnecessary removal of viable dermis. During the last decade, hydrosurgery has become popular in burn surgery as an alternative to sharp tangential. The Versajet™ hydrosurgery system (Smith and Nephew, St. Petersburg, FL, USA) was developed in 1997 for the purpose of debriding many types of wounds, including burn prior to skin grafting. This system uses a high-pressure jet of sterile physiological saline to debride wounds, drawing tissue debris and fluid into a chamber via the Venturi effect created by the saline jet. Using the cutting effect of the high-pressure jet of saline, tissue may theoretically be excised in a tangential manner with maximal dermal preservation. This preservation of dermal tissue, which might have been excised by conventional techniques, suggests that subsequent scarring might be reduced. Two randomised controlled trials compared conventional and hydrosurgical excision of burns^1,2^. However, although scar quality is considered to be one of the most important outcomes in burn surgery, these studies did not address scar quality as primary outcome. One retrospective study did compare scar quality as primary long term outcome, and reported a better scar quality after hydrosurgical excision in a subgroup (age 5 years and older) of their study population 1 year post surgery^3^. On the other hand, Hyland et al. (2007) reported no significant differences found between scarring at 3 or 6 months after-injury as a secondary outcome in a prospective RCT comparing conventional excision with hydrosurgical excision^2^. To evaluate the

added value of hydrosurgery, a prospective randomised clinical trial is needed.

**Objective**: To assess long term scar quality of deep dermal burns after debridement with hydrosurgical excision versus conventional tangential excision.

**Study design:** A multicentre intra-patient randomized controlled trial.

**Study population:** All burn patients admitted to one of the three Dutch burn centres with deep dermal burns of at least 50cm^2^ who require excision and grafting will be included. In total 105 patients will be recruited with a follow-up of 12 months post-surgery.

**Intervention:** Prior to surgery two intervention areas will be appointed, which are randomly allocated to either hydrosurgical- or conventional tangential excision. Peroperatively, two punch biopsies in each intervention area will be collected before and after excision to evaluate the debridement efficacy and to determine dermal preservation.

**Main study parameters/endpoints:** The main study parameter is scar quality 12 months post-surgery as assessed with the POSAS (Patient and Observer Scar Assessment Scale) scored independently by two blinded observers. In addition, we will compare other scar quality outcomes 3, 6, and 12 months post-surgery and dermal preservation after the two different kinds of surgery. Alongside the trial we will perform a clinimetric study into the Minimal Important Change of the POSAS. To this end we will document the global perceived effect of both excision techniques.

**Nature and extent of the burden and risks associated with participation, benefit and group relatedness:** Both treatments are applied in the Netherlands and considered standard treatment. Therefore, there is no additional risk or discomfort for the patient.

Two punch biopsies of both intervention areas will be collected before and after excision during surgery when the patient is sedated. In this trial we use the smallest punch biopsy with a diameter of 3 mm, so interference is limited e since a better scar quality is the aim of this study. As the assessment of scar outcomes by the Patient and Observer Scar Assessment Scale, Cutometer® and Dermaspectometer® test are all non-invasive measurements requiring limited registration time, the total duration of all measurements is estimated at 30 minutes per subject. No additional risks are to be expected. When possible, scar quality is assessed combined with a regular outpatient visit to the burn centre physician. If patients are willing to participate but unable to visit the outpatient department, the patient will be visited by members of the research team.

# INTRODUCTION AND RATIONALE

Surgical debridement is an important step in the treatment of burn patients. Its purpose is to remove necrotic and/or infectious tissue and to prepare the wound for definitive healing. Conventional surgical debridement of burn wounds prior to skin grafting commonly consists of sharp tangential excision of non-viable burn eschar with hand-held knives. This procedure is not only associated with substantial blood loss, but also with unnecessary removal of viable dermis^2,4-6^. During the last decade, hydrosurgery, a new alternative type of tangential excision, has become popular in burn surgery. The Versajet™ hydrosurgery system (Smith and Nephew, St. Petersburg, FL, USA) was developed in 1997 for the purpose of debriding many types of wounds, including burns prior to skin grafting. The Versajet II system was CE marked in 2011 and was launched in 2012^7^. The Versajet II™ system works by producing a high-pressure jet of water across an aperture in an angled handpiece. The Venturi effect creates a vacuum that removes surface debris, which is sucked into the machine together with the irrigation fluid. The cutting and aspiration effects can be controlled by adjusting console power settings, handpiece orientation, and handpiece pressure. Tissue may theoretically be excised in a tangential manner with maximal dermal preservation. This preservation of dermal tissue, which might have been excised by conventional techniques, suggests that subsequent scarring might be reduced.

Because burn specialists assume superior results when using hydrosurgery in the treatment of deep dermal burns, it is a widely accepted alternative for conventional tangential debridement.

In the last 6 years in Dutch burn care, each year around 750 patients were hospitalised in one of the three burn centres. Of this number approximately 40% needed surgical treatment for their burn injuries. In over 80% of the operations tangential excision was the preferred technique. In 40% of these operations hydrosurgery was used for tangential excision and conventional excision with a knife for the remaining 60%^8^. However, a limited amount of studies is available on the effects of hydrosurgery.

Several clinical studies described the efficacy and safety of the Versajet hydrosurgery apparatus as a wound debridement tool in paediatric and adult burns^5,9-13^. There are four known prospective randomised controlled trials published in the medical literature comparing Versajet™ with conventional debridement in acute and chronic wounds^1,2,14,15^.

Up to now, studies of Gravante et al. (2007) and Hyland et al. (2015) have been the only published randomised controlled trials that compared Versajet™ and conventional debridement in burn patients^1,2^. The conclusions of both studies were that there were no significant differences in healing time, contracture rates or post-operative pain between either treatment methods.

Although scar quality is considered to be one of the most important outcomes in burn surgery, both studies did not address scar quality as primary outcome.

Gravante et al. found a shorter time of debridement for difficult areas such as the face, hands and genitalia^1^. However, the overall operation duration was equivocal. They described that in all patients who were treated with the Versajet™ system adequate debridement of the wound bed was possible during surgery. They suggested that hydrosurgical tangential excision was more precise in obtaining the correct dermal plane following debridement. They did not confirm this with objective measurement tools and they had no follow-up of patients to report scar quality. Only Hyland et al. reported histological evidence to support a significant difference in the amount of viable dermal preservation between the two groups in favour of the hydrosurgical group^2^. As a secondary outcome Hyland et al. did not observe significant differences in short term scar quality measured with the Vancouver Scar Scale at 3 and 6 months. Unfortunately, this study did not objectively assess scarring. Also, data on long term follow-up were unavailable, which is essential as scars mature over a period of at least one year^16,17^. Nevertheless, a retrospective study conducted at the Burn Center of the Martini Hospital of Groningen did compare scar quality as primary long term outcome, using the observer score of the POSAS (Patient and Observer Scar Assessment Scale) which is a subjective assessment tool. They reported a better scar quality after hydrosurgical excision in a subgroup (age 5 years and older) of their study population^3^. To assess the importance of hydrosurgery on quality of burn scar outcome we want to conduct a prospective randomised clinical trial.

The primary objective of this study is to compare long term scar quality of deep dermal burns after debridement with hydrosurgical- and conventional tangential excision.

In this study, we will use several objective and subjective tools for standardized scar evaluation. General aspects of the scar are evaluated by the POSAS.

Our secondary objectives are 1) To compare dermal preservation following tangential excision either performed hydrosurgically or conventionally and 2) To determine the minimal clinical important (MIC) change of the Patient and Observer Scar Assessment Scale (POSAS). The POSAS has proven to be a reliable, feasible and valid scar assessment scale. As clinimetric development is ongoing, a value for the MIC (minimal important change) is still lacking. Without a MIC, it is not possible to translate POSAS scores into clinical important and relevant differences for patients^18,19^. Especially in this study, in which the expected differences in scar quality between the two excision techniques may be subtle, a MIC value is crucial for interpreting study results. For this reason, we will conduct a clinimetric sub study alongside this RCT to determine the MIC of the POSAS. Additionally, we will determine the MCID (Minimal clinically important difference) of the POSAS at final follow up. This is the minimal difference in scar quality that patients experience as important.

# OBJECTIVES

**Primary objective:**

- To assess superior long term scar quality of deep dermal burns after debridement with hydrosurgical versus conventional tangential excision.

**Secondary objectives:**

- To demonstrate increased dermal preservation in deep dermal burns after debridement with hydrosurgically versus conventional excision.
- To determine the minimal clinical important (MIC) change of the Patient and Observer Scar Assessment Scale (POSAS).

# STUDY DESIGN

This study will be a multicentre randomized intra-patient controlled trial with a study period of 3 years. The burn centers of the Red Cross hospital in Beverwijk, the Martini hospital in Groningen and the Maasstad hospital in Rotterdam will participate.

Patients treated in the burn centre of the Red Cross hospital, Martini hospital, and Maasstad hospital between 02/01/2017 and 01/07/2018

Patients excluded from prospective cohort:

- Burn wounds < 50cm^2^
- TBSA > 30%
- Full thickness burns
- Chemical or electrical burns
- Infected wounds
- Patients that are unlikely to comply with requirement of the study protocol and follow-up
- No informed consent

Patients eligible for trial

(Deep dermal burned and an indication for hydrosurgical or conventional tangential excision)

Study patients included

Debridement with hydrosurgery and conventional excision

Loss to follow-up

3 months follow-up

Scar measurements and questionnaires

Loss to follow-up

6 months follow-up

Scar measurements and questionnaires

Loss to follow-up

12 months follow-up

Scar measurements and questionnaires

# STUDY POPULATION

## Population (base)

All burn patients admitted to the Beverwijk, Groningen or Rotterdam Burn Centre with deep dermal burns who require excision and grafting.

## Inclusion criteria

- - - All ages
    - Deep dermal burns who require primary excision and grafting
    - Informed consent

## Exclusion criteria

- Burn wound areas <50 cm^2^
- Full thickness burns
- Chemical or electrical burns
- Infected wounds (clinical symptoms in combination with positive wound swabs)
- Insufficient knowledge of the Dutch or English language
- Patients that are unlikely to comply with requirement of the study protocol and follow-up

## Sample size calculation

Power calculation is based on the result obtained by Scholten et al.^3^ in their retrospective study on scar quality after hydrosurgery versus guarded knife excision.

The mean POSAS Observer Score in the hydrosurgery was 14.7 versus 16.7 in the guarded knife group 12 months post-surgery, with a pooled SD of 6.53, resulting in an effect size of 0.3. Because of the within-subject design, a correction for correlated samples was included, assuming a correlation of 0.4 between POSAS Observer Score within one patient. Given a power of 0.8 and a level of significance of 0.05 a number of 105 patients is needed. Because our primary outcome is not assessed until one year post-surgery we anticipate on a drop out of 30%, which means we will include 137 patients.

Annually approximately 750 patients are admitted to one of the three Dutch burn centres. From these patients approximately 300 will require a surgical intervention of which 240 consists of tangential excision. We expect that the majority of these patients, but at least 144 patients (60%), will be eligible for this study. From these eligible patients we expect 108 patients (75%) to give informed consent to participate in the study per year. So, we expect to be able to include 137 patients in 16 months.

# TREATMENT OF SUBJECTS

## Investigational product/treatment

Prior to surgery two study areas of treatment will be marked and documented by photography. Subsequently the areas will be randomly allocated to either hydrosurgical- or conventional tangential excision.

Two punch biopsies of both intervention areas will be collected before and after excision. This leads to the total number of 4 punch biopsies per patient. Type of mesh graft and expansion will also be standardised (identical mesh enlargement and symmetric covering) to ensure an equal mesh cover of the two intervention areas. Before and after excision and biopsies, surgery and topical care will be as usual.

## Use of co-intervention (if applicable)

Not applicable

## Escape medication (if applicable)

Not applicable

# INVESTIGATIONAL PRODUCT

## Name and description of investigational product(s)

**The Versajet™ II hydrosurgery system (Smith and Nephew. St. Petersburg, FL, USA)** was CE marked in 2011 and was launched in 2012^7^. It uses a high-pressure jet of sterile normal saline to debride wounds. It is attached to a console, which is then operated by a foot pedal. Physiological saline is forced out of a narrow nozzle. This jet of pressurised normal saline functions like a knife and the hand piece allows debridement and aspiration of debris to occur simultaneously. Pressure can be adjusted to facilitate the desired depth of debridement^7^.

Figure 1. The Versajet™ hydrosurgery system

[
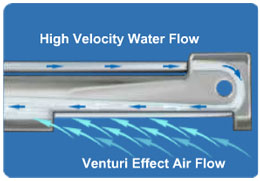
](http://www.google.nl/url?sa=i&rct=j&q=&esrc=s&source=images&cd=&cad=rja&uact=8&ved=0ahUKEwjq1OPZnf_OAhWODBoKHX5LBNoQjRwIBw&url=http://aghaii.persianblog.ir/post/51/&psig=AFQjCNHD-mFalGjg-_jtoC1VC6bsRxy5eQ&ust=1473405568021263) [
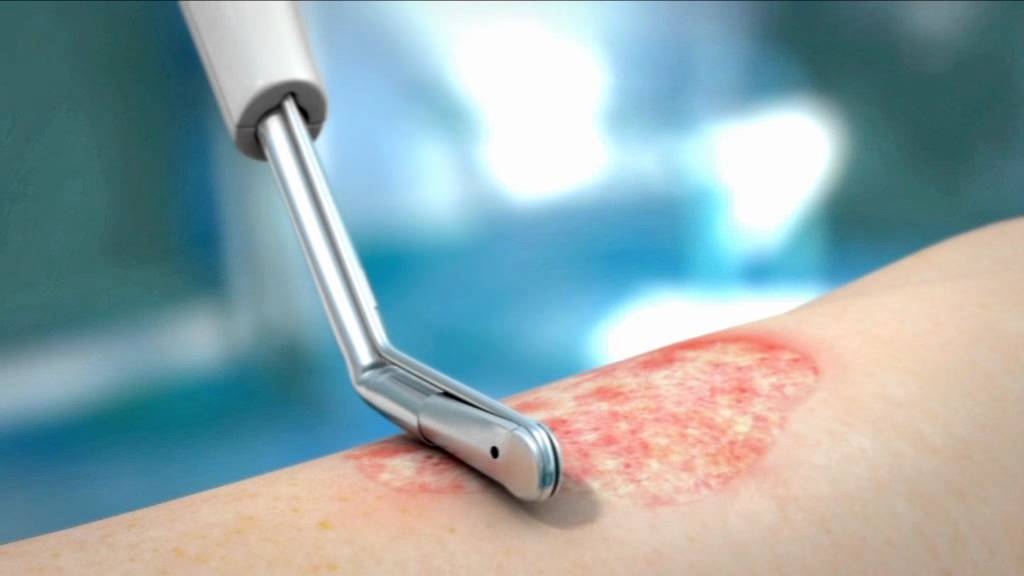
](https://www.google.nl/url?sa=i&rct=j&q=&esrc=s&source=images&cd=&cad=rja&uact=8&ved=0ahUKEwjXzaSDr__OAhUHfRoKHYXAACIQjRwIBw&url=https://www.youtube.com/watch?v=Tq7l6fDF4qw&psig=AFQjCNH7zFKKefknUWQ6-JYbcTDVhcIQlg&ust=1473410231718655)

**Tangential blade excision** relies on the stepwise excision of a layer of tissue using a flat blade, which used to be unguarded. The addition of a guard prevents the removal of excessive amounts of tissue, and most of these knives allow adjustment of the width of the gap between the blade and the guard. If the gap is too narrow, the instrument will glide off the burn without any debridement taking place. Tangential blade excision allows the excision of slithers of tissue until healthy tissue is reached^20^.

During surgery two **punch biopsies** will be taken at both intervention areas pre- and post-excision, using a 3 mm punch, in order to confirm depth of burn. The biopsies will be processed according to a standard protocol. The biopsy will be formalin fixed and processed into 5-μm histological slides. Sections will then be stained with haematoxylin and eosin (H&E). Wound depth, dermal preservation and debridement efficacy will be measured by digital imaging analysis per biopsy.

## Summary of findings from non-clinical studies

In patients with an indication for surgical treatment of their burns admitted in one of the three Dutch burn centres in the last 6 years approximately 40% needed surgical treatment for their burn injuries. In over 80% of the operations tangential excision was the preferred technique: hydrosurgery (Versajet) in 40% and conventional excision in 60% of the cases^8^.

## Summary of findings from clinical studies

Surgical debridement is an important step in the treatment of burn patients. Its purpose is to remove necrotic and/or infectious tissue and to prepare wounds for definitive healing.

The Versajet II™ hydrosurgery system is developed for the purpose of debriding many types of wounds, including burns prior to skin grafting. It works by producing a high-pressure jet of water across an aperture in an angled handpiece. The Venturi effect creates a vacuum that removes surface debris, which is sucked into the machine together with the irrigation fluid. The cutting and aspiration effects can be controlled by adjusting console power settings, handpiece orientation, and handpiece pressure. Tissue may theoretically be excised in a tangential manner, with maximal dermal preservation. This preservation of dermal tissue, which may have been excised by conventional means, suggests that subsequent scarring might be reduced. Conventional surgical debridement of acute burn wounds prior to skin grafting commonly consists of sharp tangential excision of non-viable burn eschar with hand-held knives. This procedure is not only associated with substantial blood loss, but also with unnecessary removal of viable dermis. During the last decade hydrosurgery has become popular in burn surgery. The clinical efficacy of the Versajet™ hydrosurgery apparatus as a burn wound debridement tool has been described in several clinical studies^12,13^ and several of these support the use of the system in children^9^. The majority of these studies have reported good clinical results with minimal adverse outcomes^5,9-13^.

There are four known prospective randomised controlled trials published in the medical literature that compared Versajet™ with conventional debridement in acute and chronic wounds^1,2,14,15^, and two of which have been undertaken in burns^1,2^. It is assumed that the clinical evidence for the Versajet system would apply also on the Versajet II system, because the manufacturer’s website cites references to studies on the Versajet system rather than any new studies on the Versajet II system^7^. Therefore, the evidence of the Versajet system is summarised.

Two prospective randomized controlled trials on the Versajet system for patients with chronic wounds have been published:

- Caputo et al. (2008) measured the debridement time with Versajet (n=19) and sharp debridement using a scalpel (n=22) in patients with lower extremity ulcers. There was significant evidence (P < 0.008) of a shorter debridement time using Versajet. They did not find a significant difference in median time to wound closure^14^
- Liu et al. (2015) reported the clinical and cost effectiveness of Versajet (n=21) in comparison to conventional debridement of chronic wounds using a scalpel (n=19). They found a significant improvement for first excision time (p 0,005) and blood loss (p 0.003), but no difference in time to wound closure or bacterial count. Also the Versajet system was calculated to be cost neutral^15^.

Several prospective randomized controlled trials on the Versajet system for patients with burns have been conducted:

*Unpublished studies:*

- Anniboletti et al. (2011) reported their experience with Versajet (n=17) vs. conventional escharectomy (n=18), including 12 children. They found a shorter healing time with an average of 7 days. There was no difference in time to debridement or healing time. This study was only presented at a conference so additional information on the study could not be found^21^.
- Esposito et al. (2009) reported their experience with Versajet (n=14) vs. conventional escharectomy (n=12), including 8 children to evaluate speed to debridement. They did not find a time difference. This study was only presented as a conference abstract^22^.

Both studies described the Versajet system to be an easier operating tool to reach the desirable dermal plane.

*Published studies:*

- Gravante et al. (2007) compared Versajet (n=42) to conventional escharectomy (n=45) in adults. They found a shorter debridement time for difficult areas such as the face, hands and genitalia. However, operating time overall was equivocal. They didn‘t find a significant difference in complete healing of the wounds, post-operative pain or contractures at 6 months^1^.
- Hyland et al. (2015) compared the Versajet system (n=30) to conventional debridement (n=31) in children (≤ 16 years). They found a histologically confirmed significant difference in the amount of viable dermal preservation between the two groups (p 0.02) with more viable tissue lost in the conventional group. They did not find a significant difference between graft take at day 10, amounts of post-operative wound infection, duration of surgery time or time to healing after grafting. There were no significant differences found between scarring at 3 or 6 months after surgery.

Although scar quality is considered to be one of the most important outcomes in burn surgery, none of these studies addressed scar quality as a primary outcome. Only Hyland et al. assessed scar quality after 3 and 6 months as a secondary outcome measure using the modified Vancouver Scar Scale. This scale is a subjective scale, only taking the observers opinion into account^23^. They didn‘t study patient perception of scars and didn’t perform any objective scar measurements. Data on long term follow-up was also unavailable which is essential as scars mature over a period of at least one year^16^.

A retrospective observational study conducted at the Burn Center of the Martini Hospital of Groningen did compare scar quality as primary long term outcome, using the observer scale of the POSAS, which is a subjective assessment tool^3^. They reported a better scar quality after hydrosurgical excision in a subgroup (age 5 years and older) of their study population. To provide stronger evidence whether hydrosurgery results in a superior scar quality, a prospective study is needed to confirm the promising results of this retrospective study.

## Summary of known and potential risks and benefits

**Tangential blade excision**: Since the blade is long, this technique will only work on a convex curved surface. Such convexity can be found in many, but not all parts of the body. Areas such as those around the hands, malleoli and the axilla are difficult to excise in this manner. These knives allow the excision of slithers of tissue until healthy tissue is reached. This technique has the advantage of being fast and can lead to the removal of too much tissue although that has no adverse effect on the skin graft. Another potential problem with tangential blade excision is that at the periphery of the excision there is inevitably some ‘shelving’ of the excision, as the depth of excision slopes from zero at the very edge to deeper once the limit of the guard is reached. As split thickness skin grafts are usually of a uniform thickness, this will lead to an excess of tissue at the periphery of the wound. This can lead to a ‘stuck on’ appearance of the skin graft.^20^

**The Versajet™ hydrosurgery system (Smith and Nephew)** in expert opinion allows a more precise removal of unhealthy tissue and has the ease of blade excision but reduces the potential for excessive, unnecessary removal of healthy tissue. This technique allows the narrowest excision margin currently available, as layers of as little as 50 μm (0.05 mm) can be removed ^20^.

In paragraph 6.3 a summary of findings from clinical studies is described. The outcomes suggested mixed results.

## Description and justification of route of administration and dosage

Not applicable

## Dosages, dosage modifications and method of administration

Not applicable

## Preparation and labelling of Investigational Medicinal Product

Not applicable

## Drug accountability

Not applicable

# NON-INVESTIGATIONAL PRODUCT

Not applicable

# METHODS

## Study parameters/endpoints

### Main study parameter/endpoint

- Scar quality assessed as the total observer score of the POSAS at 12 months post-surgery scored by 2 blinded observers independently^24,25,25^

### Secondary study parameters/endpoints (if applicable)

- Dermal preservation (2 punch biopsies pre- and post-excision).
- POSAS scores: a validated questionnaire designed for subjective evaluation of

various types of scar formation^25,26^ (see paragraph 8.3)

- Global perceived change: the anchor question of the POSAS, to be able to assess the minimal clinical important change and minimal clinically important difference of the POSAS (see Statistical analysis, p.32)^18,19^
- Scar elasticity measured with the Cutometer®, a validated instrument. It measures the vertical deformation of the skin in millimetres when the skin is pulled by means of a controlled vacuum into a circular aperture^27^. (see paragraph 8.3)
- Scar colour and pigmentation measured with the Dermaspectrometer®. This is a validated instrument to measure scar colour by a narrow-band simple reflectance meter ^28^. (see paragraph 8.3)

### Other study parameters (if applicable)

- Demographics: age, sex
- Burn characteristics: % total burned body surface area (TBSA), anatomical affected site(s), date of injury, burn depth determined by an experienced burn physician and burn depth determined by a LDI-scan on date 2-5 post burn (prior to the operation), date of surgery.
- Clinical characteristics: Fitzpatrick skin type, timing to surgery, wound healing time (measured in days till 95% re-epithelization), determination of bacterial load, comorbidity, weck knife, dermatome and Versajet settings, expansion of skin graft , adverse events (blood loss, graft loss, wound infection) and need for reconstructive surgery.

## Randomisation, blinding and treatment allocation

Randomisation will be at intra-patient level. In each patient two intervention areas will be appointed, and defined A and B. The allocation of the treatment will be at random using the online randomisation program CASTOR, <https://data.castoredc.com>. In surgical treatment blinding is not possible, as the burn surgeon knows which part of the wound receives what surgical treatment. After randomisation the local principal investigator and the central trial co-ordinator will receive an email with the inclusion number and the randomisation outcome. The outcome will be displayed on the website as well, only visible for the central trial co-ordinator. Outcome assessment will be blinded as the junior investigator/research nurse who does the follow-up measurements is unaware of the technique used in areat A or area B.

In case of randomisation related difficulties the central trial co-ordinator or research coordinator can be contacted.

## Study procedures

**Peroperatively**

After receiving adequate information and when informed consent is signed two study areas of equal burn deapth are marked as area A and area B (figure 2). This must be documented by photography prior to surgery. We will take a 3 mm punch biopsy from part A and part B (figure A, dot A and B). After the biopsies we randomly allocate part A and B to hydrosurgical- or conventional excision. After-debridement we will take one more punch biopsy from both wound areas (figure 2, dot C and D), adjacent to the previous punch biopsy site in an area of confluent depth, prior to application of a split thickness skin graft. Type of mesh graft and expansion will be standardized to ensure an equal mesh cover of the two intervention areas.

Figure 2. Location of punch biopsies.


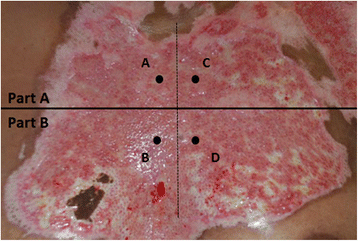


In this trial we use the smallest punch biopsy with a diameter of 3 mm, as interference is limited since a better scar quality is the aim of this study. Wound tissue is collected before and after excision in both intervention areas, which leads us to the total number of 4 punch biopsies per patient.

The stained biopsy specimens will be analysed using light microscopy. To determine the amount of dermal preservation and hence precision of debridement, the amount of viable tissue on pre- and post-debridement specimens will be recorded.

**Post-surgery and follow-up**

Patients will be assessed in both the acute phase and during the outpatient follow-up. We will record all complications during and post-surgery and the time to wound closure (95% re-epithelization). Blinded follow-up measurements will be performed at 3, 6 and 12 months post-surgery by trained research nurses and/or junior researchers. These study follow-up visits will take place in the outpatient clinic and the timing is similar to the standard follow up schedule after a burn injury in Dutch specialized burn care.

During this visit the POSAS, anchor questions (global perceived effect) of the POSAS, the Cutometer® and DermaSpectrometer® measurements will be administered and whether the patient has had reconstructive surgery. All study wounds and scars will be documented by photography before excision and 3,6 and 12 months after excision.

*Subjective scar assessment*

Patient and Observer Scar Assessment Score (POSAS)

The Patient and Observer Scar Assessment Scale (POSAS) will be used to assess the scar quality of part A and part B. This validated questionnaire enables both patient and observers to assess the same scar on six different scar characteristics, with roughly an overlap of four characteristics. The patient assesses the scar on pain, pruritus, colour, thickness, surface roughness and pliability, while the observer assesses the scar on vascularity, pigmentation, thickness, relief, pliability surface study areas A and B. Both use a numerically 10-point scale in which 1 represents a scar comparable with ‘normal skin’ while 10 represents the ‘worst scar imaginable’. Both patient and observer independently assess the same scar. The observer assessment will be performed by an experienced and trained observer, either a physician, nurse or researcher. ^23,29^

Anchor question of the POSAS

The anchor is a question regarding change in perceived scar quality in the intervening periods between two assessments and can be answered on a 5-point Likert scale. This scale has the following response options: much better/slightly better/no change/slightly worse/much worse and will be added to the POSAS used for the 6 and 12 months follow-up assessment. Another anchor question is about whether patients think that the scar of area A is better than the scar of area B or vice versa. Again, response options can be chosen on a 5-point Likert scale with the following options: much better/slightly better/no change/slightly worse/much worse.

*Objective scar assessment*

The measurements with the Cutometer® and Dermaspectrometer® have to be performed on fixed locations in order to prevent selection bias in collecting the objective data. They will be performed on 5 locations following an algorithm in which scars are divided by two lines from the widest length and width through the centre of the scar (diamond shape). The 5 points are chosen halfway between these lines and in the centre. All measurements will be done this way separately in study area A and study area B of the wound. This algorithm is used in different other studies and is illustrated in the figure 3 below ^30,31^.


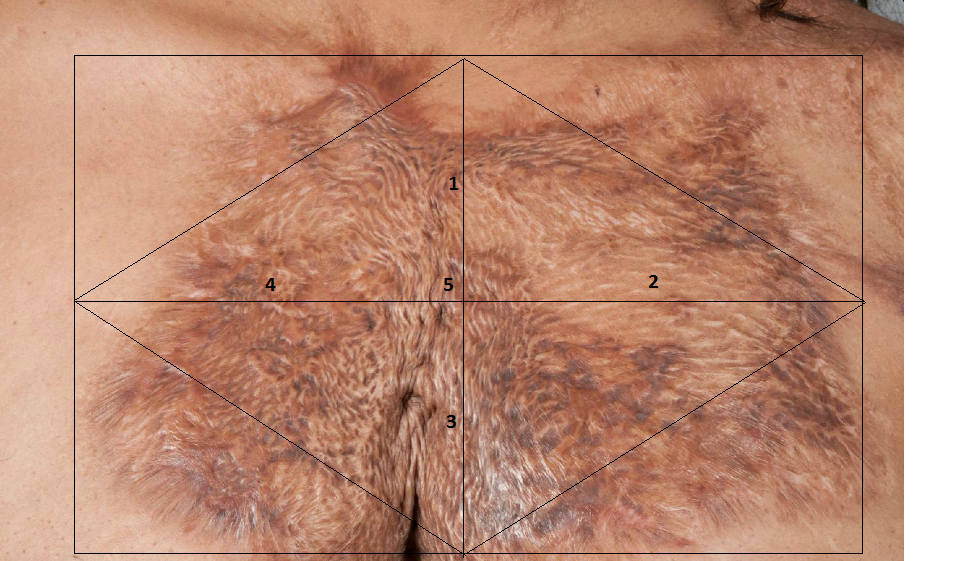


Figure 3. Location of the 5 measurements

Cutometer**®** Skin Elasticity Meter 575
The Cutometer® (Courage-Khazaka electronic GmbH Cologne, Germany) is a non-invasive suction device that has been applied for the objective and quantitative measurement of skin elasticity. It measures the viscoelasticity of the skin by analysing its vertical deformation in response to negative pressure^23^. The Cutometer® provides several elasticity parameters. In case of substantial heterogeneity in individual scars, these will be measured following a standardised method, including 5 scar (figure 3) and 2 control measurements. The first option for the control measurements will always be the patients’ contralateral site. In case the contralateral site is also affected, the most comparable and unaffected spot near the scar will be used.

DermaSpectrometer

The Dermaspectrometer® (Cortex Technology ApS Hadsund, Denmark) will be used to characterize vascularity and pigmentation and thus the colour of scars/skin. This instrument’s light emitting diodes emit light at two defined wavelengths: 568 nm (green) and 655 nm (red). A photo detector measures the light reflected by the skin. It measures the absorbed and reflected light at wavelengths, respectively, in the green and the red for haemoglobin and for melanin. A melanin index and an erythema index are computed from the intensity of the absorbed and the reflected light at, respectively, 568 and 655 nm^25^. All scars will be measured following a standardised method, including 5 scar (figure 3) and 1 control measurement. The first option for the control measurement is patients contralateral side. When this is not possible the measurement must be done on an adjacent location.

To analyse the indices for erythema and melanin the absolute values of the subtraction of the scar and normal skin will be used.

In table 1 a summary of the study procedures is presented.

**Table 1. Study procedures**

|  | **Before inclusion** | **On admission**  **-** Treatment phase | **Follow-up** | *(months)* |  |
| --- | --- | --- | --- | --- | --- |
|  |  |  | 3 | 6 | 12 |
| **Standard screening, baseline procedures** | **x** |  |  |  |  |
| **Check eligibility (inclusion criteria)** | **x** |  |  |  |  |
| **Patient Information** | **x** |  |  |  |  |
| **Obtain written informed consent** | **x** |  |  |  |  |
| **Randomisation** |  | **x** |  |  |  |
| **Photography** |  | **x** *- During surgery* |  |  |  |
| **Biopsies** |  | **x** *- During surgery* |  |  |  |
| **Re-epithalisatie 95% (days)** |  | **x** |  |  |  |
| **Complications** |  | **x** *- Per-operatively and post-operatively* |  |  |  |
| **Cutometry** |  |  | **x** |  | **x** |
| **Dermaspectrometry** |  |  | **x** |  | **x** |
| **Questionnaires**  - POSAS  - Anchor questions |  |  | **x x** | **x x** | **x x** |
| **Biopsy assessment** |  |  |  |  | **x** |

## Withdrawal of individual subjects

Subjects can leave the study at any time for any reason if they wish to do so without any consequences. The investigator can decide to withdraw a subject from the study for urgent medical reasons.

### Specific criteria for withdrawal (if applicable)

Not applicable

## Replacement of individual subjects after withdrawal

There will be no replacement of individual subjects after withdrawal.

## Follow-up of subjects withdrawn from treatment

Subjects withdrawn from the study will receive every treatment as usual and all the follow ups and after care as foreseen for every other burn patient.

## Premature termination of the study

No criteria are foreseen for premature termination of the study.

# SAFETY REPORTING

## Temporary halt for reasons of subject safety

In accordance to section 10, subsection 4, of the WMO, the sponsor will suspend the study if there is sufficient ground that continuation of the study will jeopardise subject health or safety. The sponsor will notify the accredited METC without undue delay of a temporary halt including the reason for such an action. The study will be suspended pending a further positive decision by the accredited METC. The investigator will take care that all subjects are kept informed.

## AEs, SAEs and SUSARs

### Adverse events (AEs)

Adverse events are defined as any undesirable experience occurring to a subject during the study, whether or not considered related to surgery using hydrosurgery (Versajet) or conventional tangential excision. All adverse events reported spontaneously by the subject or observed by the investiga­tor or his staff will be recorded.

### Serious adverse events (SAEs)

A serious adverse event is any untoward medical occurrence or effect that

- results in death;
- is life threatening (at the time of the event);
- requires hospitalisation or prolongation of existing inpatients’ hospitalisation;
- results in persistent or significant disability or incapacity;
- is a congenital anomaly or birth defect; or
- any other important medical event that did not result in any of the outcomes listed above due to medical or surgical intervention but could have been based upon appropriate judgement by the investigator.

An elective hospital admission will not be considered as a serious adverse event.

The sponsor will report the SAEs through the web portal *ToetsingOnline* to the accredited METC that approved the protocol, within 7 days of first knowledge for SAEs that result in death or are life threatening followed by a period of maximum of 8 days to complete the initial preliminary report. All other SAEs will be reported within a period of maximum 15 days after the sponsor has first knowledge of the serious adverse events.

### Suspected unexpected serious adverse reactions (SUSARs)

Adverse reactions are all untoward and unintended responses to an investigational product related to any dose administered.

Unexpected adverse reactions are SUSARs if the following three conditions are met:

1. the event must be serious (see chapter 9.2.2);
2. there must be a certain degree of probability that the event is a harmful and an undesirable reaction to the medicinal product under investigation, regardless of the administered dose;
3. the adverse reaction must be unexpected, that is to say, the nature and severity of the adverse reaction are not in agreement with the product information as recorded in:

- Summary of Product Characteristics (SPC) for an authorised medicinal product;
- Investigator’s Brochure for an unauthorised medicinal product.

The sponsor will report expedited the following SUSARs through the web portal *ToetsingOnline* to the METC

- SUSARs that have arisen in the clinical trial that was assessed by the METC;
- SUSARs that have arisen in other clinical trials of the same sponsor and with the same medicinal product, and that could have consequences for the safety of the subjects involved in the clinical trial that was assessed by the METC.

The remaining SUSARs are recorded in an overview list (line-listing) that will be submitted once every half year to the METC. This line-listing provides an overview of all SUSARs from the study medicine, accompanied by a brief report highlighting the main points of concern.

The expedited reporting of SUSARs through the web portal Eudravigilance or ToetsingOnline is sufficient as notification to the competent authority.

The sponsor will report expedited all SUSARs to the competent authorities in other Member States, according to the requirements of the Member States.

The expedited reporting will occur not later than 15 days after the sponsor has first knowledge of the adverse reactions. For fatal or life threatening cases the term will be maximal 7 days for a preliminary report with another 8 days for completion of the report.

## Annual safety report

In addition to the expedited reporting of SUSARs, the sponsor will submit, once a year throughout the clinical trial, a safety report to the accredited METC, competent authority, and competent authorities of the concerned Member States.

This safety report consists of:

- a list of all suspected (unexpected or expected) serious adverse reactions, along with an aggregated summary table of all reported serious adverse reactions, ordered by organ system, per study;
- a report concerning the safety of the subjects, consisting of a complete safety analysis and an evaluation of the balance between the efficacy and the harmfulness of the medicine under investigation.

## Follow-up of adverse events

All AEs will be followed until they have abated, or until a stable situation has been reached. Depending on the event, follow up may require additional tests or medical procedures as indicated, and/or referral to the general physician or a medical specialist.

SAEs need to be reported till end of study within the Netherlands, as defined in the protocol

## Data Safety Monitoring Board (DSMB) / Safety Committee

Not applicable

# STATISTICAL ANALYSIS

Data analysis will be performed using SPSS PASW Statistics 23.0 (IBM, New York City). Normality of continuous data will be tested with the Shapiro-Wilk and Kolmogorov-Smirnov test and by inspecting frequency distributions histograms.

## Primary study parameter(s)

Scar quality 12 months post-surgery assessed by the observer score of the POSAS scale will be tested for normality before univariate analyses will be performed. If the data are normally distributed, a paired student’s t-test will be used for comparison of the data. Otherwise the Wilcoxon signed-rank test will be applied.

## Secondary study parameter(s)

Differences in viable dermis after excision (measured by histopathology), differences in time to wound healing (time to 95% re-epithalisation in days), scar elasticity (measured by the Cutometer®) scar vascularisation and pigmentation (measured by the DermaSpectrometer®) will be analysed using the Wilcoxon signed-rank test or paired student t-test if the data are normally distributed.

The MIC will be determined following the anchor-based MIC distribution method^32^. The anchor is a question regarding change in perceived scar quality in the intervening periods between two assessments and can be answered on a 5-point Likert scale. The MIC will be calculated in patients who slightly improved between three and six months post-surgery (longitudinal). We will determine the MCID (minimal clinical important difference) of the POSAS using the mean change method. This will happen at final follow-up (cross-sectional) at one year post-surgery. The anchor question is about whether patients think part A of the scar is better than part B or vice versa. Response options can be chosen on a 5-point Likert scale. The MCID is equivalent to the mean change in score of patients who think part A of the scar is slightly better than part B or vice versa.

## Other study parameters

Not applicable

## Interim analysis (if applicable)

Not applicable

# ETHICAL CONSIDERATIONS

## Regulation statement

This study will be conducted according to the principles of the Declaration of Helsinki (Seoul, 22-10-2008) and in accordance with the Medical Research Involving Human Subjects Act (WMO) and the valid Dutch laws.

## Recruitment and consent

Patients will be recruited in the Dutch Burn Centres in Beverwijk, Groningen and Rotterdam. The patients will be informed about the content of the study, intensity for the patients and total study time by a medical researcher. The researcher as well as the patients have to sign the informed consent. Patients or their legal representatives have at least 24 hours to consider their decision. For questions they can contact the local principal investigator or the independent physician

## Objection by minors or incapacitated subjects (if applicable)

The code of conduct minors is applicable.

## Benefits and risks assessment, group relatedness

Patients experience no additional risks since debridement with hydrosurgical- and conventional tangential excision are both standard treatments.

As the POSAS, Cutometer and Dermaspectometer are non-invasive measurements requiring limited registration time the total duration of all measurements is estimated at 30 minutes per subject. No additional risks are to be expected.

## Compensation for injury

The sponsor/investigator has a liability insurance which is in accordance with article 7, subsection 6 of the WMO.

The sponsor (also) has an insurance which is in accordance with the legal requirements in the Netherlands (Article 7 WMO and the Measure regarding Compulsory Insurance for Clinical Research in Humans of 23th June 2003). This insurance provides cover for damage to research subjects through injury or death caused by the study.

1. € 450.000,-- (i.e. four hundred and fifty thousand Euro) for death or injury for each subject who participates in the Research;
2. € 3.500.000,-- (i.e. three million five hundred thousand Euro) for death or injury for all subjects who participate in the Research;
3. € 5.000.000,-- (i.e. five million Euro) for the total damage incurred by the organisation for all damage disclosed by scientific research for the Sponsor as ‘verrichter’ in the meaning of said Act in each year of insurance coverage.

The insurance applies to the damage that becomes apparent during the study or within 4 years after the end of the study.

## Incentives (if applicable)

Participants receive no special incentive, compensation or treatment by participating in this study.

# ADMINISTRATIVE ASPECTS, MONITORING AND PUBLICATION

## Handling and storage of data and documents

Data will be documented using an online CRF (Castor EDC, Ciwit BV, Amsterdam, The Netherlands). Castor EDC has been audited on GCP compliance by Profess Medical Consultancy and has obtained a GCP compliance certificate.

The CRF and study data are coded by

- - a letter B, R or G for the burn centre;
  - a study code: HyCon
  - number of patient in the study;

eg: B - HyCon - 001.

The key of the code is provided to the local principal investigators.

The relevant data will be converted to a large table in SPSS to enable statistical analyses.

Data will be handled and stored anonymously and not traceable to individual subjects. Source data will be accessible for the local investigators, coordinating investigator and statistician, and if necessary for the members of the Medical Ethics Committee and the Health Care Inspectorate (IGZ). The handling of personal data will comply with the Dutch Personal Data Protection Act. Data will be stored for 15 years.

## Monitoring and Quality Assurance

Monitoring will be conducted by the scientific bureau of the Maasstad Hospital

## Amendments

Amendments are changes made to the research after a favourable opinion by the accredited METC has been given. All amendments will be notified to the METC that gave a favourable opinion.

A ‘substantial amendment’ is defined as an amendment to the terms of the METC application, or to the protocol or any other supporting documentation, that is likely to affect to a significant degree:

- the safety or physical or mental integrity of the subjects of the trial;
- the scientific value of the trial;
- the conduct or management of the trial; or
- the quality or safety of any intervention used in the trial.

All substantial amendments will be notified to the METC and to the competent authority.

Non-substantial amendments will not be notified to the accredited METC and the competent authority, but will be recorded and filed by the sponsor.

## Annual progress report

The sponsor/investigator will submit a summary of the progress of the trial to the accredited METC once a year. Information will be provided on the date of inclusion of the first subject, numbers of subjects included and numbers of subjects that have completed the trial, serious adverse events/ serious adverse reactions, other problems, and amendments.

## Temporary halt and (prematurely) end of study report

The investigator/sponsor will notify the accredited METC of the end of the study within a period of 8 weeks. The end of the study is defined as the last patient’s last visit.

The sponsor will notify the METC immediately of a temporary halt of the study, including the reason of such an action. In case the study is ended prematurely, the sponsor will notify the accredited METC within 15 days, including the reasons for the premature termination. Within one year after the end of the study, the investigator/sponsor will submit a final study report with the results of the study, including any publications/abstracts of the study, to the accredited METC.

## Public disclosure and publication policy

The funders had no role in study design, data collection and analysis, decision to publish, or preparation of the manuscript.

# STRUCTURED RISK ANALYSIS

## Potential issues of concern

Not applicable

## Synthesis

Patients experience no additional risks since debridement with hydrosurgical- and conventional tangential excision are both standard treatments.

# REFERENCES

1. Gravante G, Delogu D, Esposito G, Montone A. Versajet hydrosurgery versus classic escharectomy for burn debridment: A prospective randomized trial. *J Burn Care Res*. 2007;28(5):720-724.

2. Hyland EJ, D'Cruz R, Menon S, et al. Prospective, randomised controlled trial comparing versajet hydrosurgery and conventional debridement of partial thickness paediatric burns. *Burns*. 2015;41(4):700-707.

3. Scholten-Jaeger S, Bosch van den M, Nieuwenhuis M. Scar quality after surgical treatment of deep dermal burns with hydro-surgery compared to quarded knife, short and long-term outcome. . ;Poster Burns: 37S:S18. 2011.

4. Cartotto R, Musgrave MA, Beveridge M, Fish J, Gomez M. Minimizing blood loss in burn surgery. *J Trauma*. 2000;49(6):1034-1039.

5. Cubison TC, Pape SA, Jeffery SL. Dermal preservation using the versajet hydrosurgery system for debridement of paediatric burns. *Burns*. 2006;32(6):714-720.

6. Gurfinkel R, Rosenberg L, Cohen S, et al. Histological assessment of tangentially excised burn eschars. *Can J Plast Surg*. 2010;18(3):e33-6.

7. NICE. the versajet II hydrosurgery system for surgical debridement of acute and chronic wounds and burns. NICE medtech innovation briefing: National institute for health care excellence; . 2014: 1-23.

8. Baar van M. Nederlandse brandwondenregistratie R3, jaarrapportage 2013. vereniging samenwerkende brandwondencentra nederland (VSBN). . 2014.

9. Klein MB, Hunter S, Heimbach DM, et al. The versajet water dissector: A new tool for tangential excision. *J Burn Care Rehabil*. 2005;26(6):483-487.

10. Sainsbury DC. Evaluation of the quality and cost-effectiveness of versajet hydrosurgery. *Int Wound J*. 2009;6(1):24-29.

11. Kimble RM, Mott J, Joethy J. Versajet hydrosurgery system for the debridement of paediatric burns. *Burns*. 2008;34(2):297-8; author reply 299.

12. Rennekampff HO, Schaller HE, Wisser D, Tenenhaus M. Debridement of burn wounds with a water jet surgical tool. *Burns*. 2006;32(1):64-69.

13. Tenenhaus M, Bhavsar D, Rennekampff HO. Treatment of deep partial thickness and indeterminate depth facial burn wounds with water-jet debridement and a biosynthetic dressing. *Injury*. 2007;38 Suppl 5:S39-45.

14. Caputo WJ, Beggs DJ, DeFede JL, Simm L, Dharma H. A prospective randomised controlled clinical trial comparing hydrosurgery debridement with conventional surgical debridement in lower extremity ulcers. *Int Wound J*. 2008;5(2):288-294.

15. Liu J, Ko JH, Secretov E, et al. Comparing the hydrosurgery system to conventional debridement techniques for the treatment of delayed healing wounds: A prospective, randomised clinical trial to investigate clinical efficacy and cost-effectiveness. *Int Wound J*. 2015;12(4):456-461.

16. van der Wal MB, Vloemans JF, Tuinebreijer WE, et al. Outcome after burns: An observational study on burn scar maturation and predictors for severe scarring. *Wound Repair Regen*. 2012;20(5):676-687.

17. Gangemi EN, Gregori D, Berchialla P, et al. Epidemiology and risk factors for pathologic scarring after burn wounds. *Arch Facial Plast Surg*. 2008;10(2):93-102.

18. de Vet HC, Terwee CB, Ostelo RW, Beckerman H, Knol DL, Bouter LM. Minimal changes in health status questionnaires: Distinction between minimally detectable change and minimally important change. *Health Qual Life Outcomes*. 2006;4:54.

19. Terwee CB, Roorda LD, Dekker J, et al. Mind the MIC: Large variation among populations and methods. *J Clin Epidemiol*. 2010;63(5):524-534.

20. Jeffery SL. Device related tangential excision in burns. *Injury*. 2007;38 Suppl 5:S35-8.

21. Anniboletti T, Palombo M, Facsciani L, Delli Santi G, Palombo S. The use of versajet hydrosurgery: 5 years experience

burns, 37 (2011), p. S19. *Burns*. 2011;37:19.

22. Esposito G, Anniboletti T, Palombo M, Palombo P. Versajet hydrosurgery: Our experience in adults and pediatric patients. *Burns*. 2009;35:23-Supl 1.

23. Fearmonti R, Bond J, Erdmann D, Levinson H. A review of scar scales and scar measuring devices. *Eplasty*. 2010;10:e43.

24. van der Wal MB, Tuinebreijer WE, Bloemen MC, Verhaegen PD, Middelkoop E, van Zuijlen PP. Rasch analysis of the patient and observer scar assessment scale (POSAS) in burn scars. *Qual Life Res*. 2012;21(1):13-23.

25. Draaijers LJ, Tempelman FR, Botman YA, et al. The patient and observer scar assessment scale: A reliable and feasible tool for scar evaluation. *Plast Reconstr Surg*. 2004;113(7):1960-5; discussion 1966-7.

26. van de Kar AL, Corion LU, Smeulders MJ, Draaijers LJ, van der Horst CM, van Zuijlen PP. Reliable and feasible evaluation of linear scars by the patient and observer scar assessment scale. *Plast Reconstr Surg*. 2005;116(2):514-522.

27. Draaijers LJ, Botman YA, Tempelman FR, Kreis RW, Middelkoop E, van Zuijlen PP. Skin elasticity meter or subjective evaluation in scars: A reliability assessment. *Burns*. 2004;30(2):109-114.

28. Draaijers LJ, Tempelman FR, Botman YA, Kreis RW, Middelkoop E, van Zuijlen PP. Colour evaluation in scars: Tristimulus colorimeter, narrow-band simple reflectance meter or subjective evaluation? *Burns*. 2004;30(2):103-107.

29. Hoogewerf CJ, van Baar ME, Middelkoop E, van Loey NE. Patient reported facial scar assessment: Directions for the professional. *Burns*. 2014;40(2):347-353.

30. van Zuijlen PP, Angeles AP, Suijker MH, Kreis RW, Middelkoop E. Reliability and accuracy of techniques for surface area measurements of wounds and scars. *Int J Low Extrem Wounds*. 2004;3(1):7-11.

31. Verhaegen PD, van der Wal MB, Bloemen MC, et al. Sustainable effect of skin stretching for burn scar excision: Long-term results of a multicenter randomized controlled trial. *Burns*. 2011;37(7):1222-1228.

32. de Vet HC, Ostelo RW, Terwee CB, et al. Minimally important change determined by a visual method integrating an anchor-based and a distribution-based approach. *Qual Life Res*. 2007;16(1):131-142.
