# Supplementary material for: Hydrosurgical and conventional debridement of burns: randomized clinical trial
Source: Br J Surg. 2022 Mar 3;109(4):332–9. doi: 10.1093/bjs/znab470 (PMC10364696; doi:10.1093/bjs/znab470)
Supplement: znab470_Supplementary_Data [file znab470_supplementary_data.zip › Supplementary_Appendix_2.docx]

**Supplementary data 2 – Specification of excluded patients**

| **Criterion** | **n**  **Total = 576** |
| --- | --- |
| **Exclusion criteria** | **504** |
| Burn wound area <50cm^2^ | 192 |
| %TBSA >30%* | 58 |
| Full-thickness burns | 124 |
| Chemical or electrical burns | 18 |
| Infected wounds | 7 |
| Insufficient knowledge of the Dutch or English language | 15 |
| Patients that were unlikely to comply with requirement of the study protocol and follow-up | 90 |
| *Psychological problems* | *34* |
| *Foreigners that returned to their homeland* | *16* |
| *Went to an highly secured prison* | *6* |
| *Unknown* | *34* |
| **Declined to participate** | **52** |
| **Other reasons** | **20** |
| Missed by the study team | 12 |
| Participation in another intervention study | 8 |
