# Supplementary material for: Hydrosurgical and conventional debridement of burns: randomized clinical trial
Source: Br J Surg. 2022 Mar 3;109(4):332–9. doi: 10.1093/bjs/znab470 (PMC10364696; doi:10.1093/bjs/znab470)
Supplement: znab470_Supplementary_Data [file znab470_supplementary_data.zip › Supplementary_Appendix_3.docx]

**Supplementary data 3 – Histological assessment**

The biopsies were fixed in kryofix, processed into 5μm histological slides and stained with a Herovici polychrome staining.^1^ A digital camera (Nikon DS-Ri2, Nikon, Amsterdam, the Netherlands) mounted on an Axioskop40FL microscope (Zeiss, Badhoevedorp, the Netherlands) was used to take images of the slides. Digital image analysis software (NIS-Elements 4.4, Nikon) was used to determine the amount of remaining dermis, measured as the length in µm between two parallel lines; one at the subcutis and one at the dermal surface (Figure 1). If the border between dermis and subcutis was not visible on the slides, or only subcutis was present, slides were excluded for analyses.


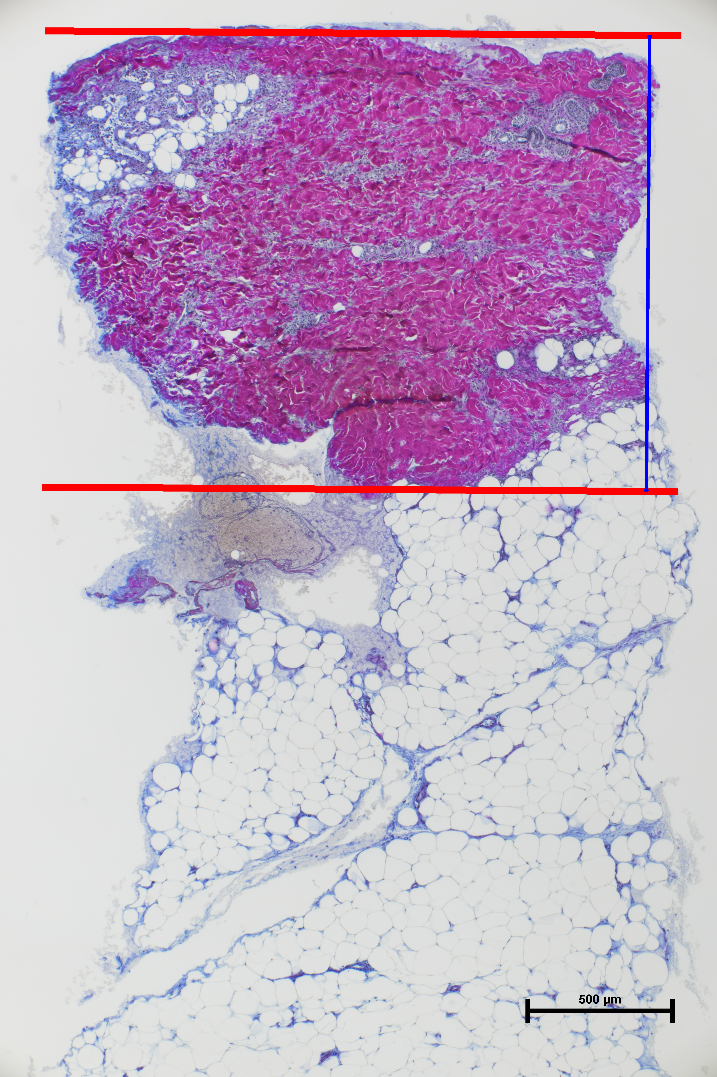

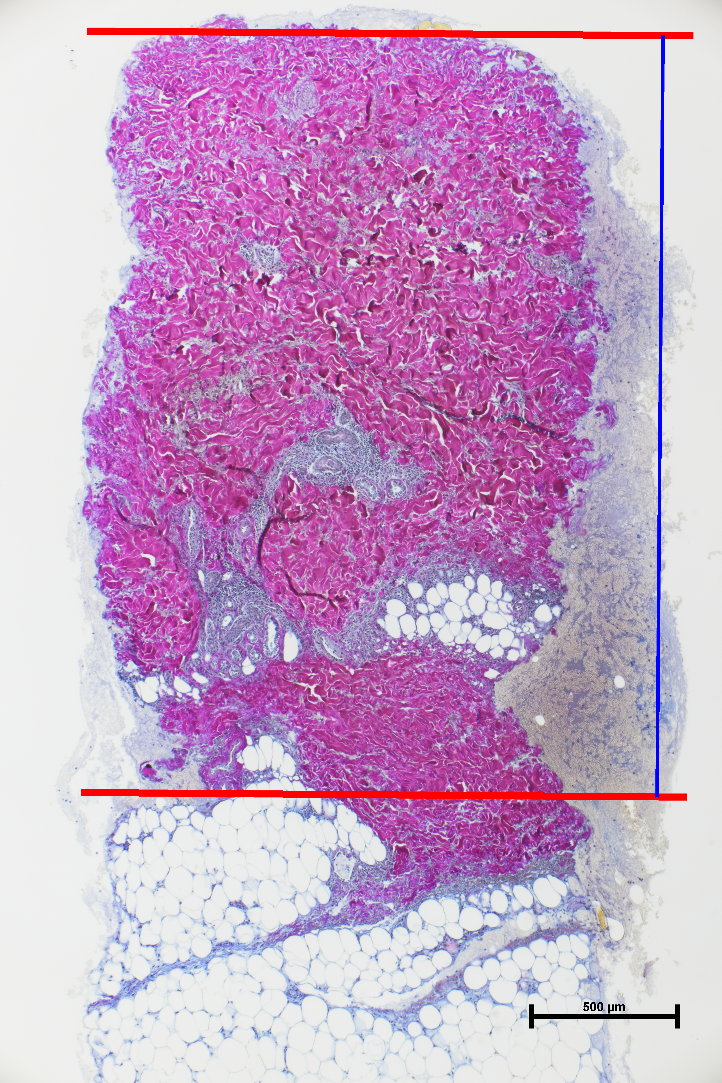
**Figure 1a and 1b.** Histological specimen demonstrating an example of Herovici polychrome staining and measurement procedure after hydrosurgical (Figure 1a, 2362 µm dermis left) and conventional (Figure 1b, 1265 µm dermis left) debridement.

**Reference**

1. Herovici C. [Picropolychrome: histological staining technic intended for the study of normal and pathological connective tissue]. *Rev Fr Etud Clin Biol* 1963;**8**: 88-89.
